# How Does Management Matter for Hospital Performance? Evidence From the Global Hospital Management Survey in China

**DOI:** 10.34172/ijhpm.8478

**Published:** 2024-12-09

**Authors:** Qinghong He, Gordon G. Liu, Jinyang Chen, Luoqi Yuan, Xuezhi Hong, Zhihua Zhang

**Affiliations:** ^1^Institute of Economics, Chinese Academy of Social Sciences, Beijing, China.; ^2^Institute for Global Health and Development, National School of Development, Peking University, Beijing, China.; ^3^China Center for Health Economic Research (CCHER), Peking University, Beijing, China.; ^4^Centre for Health Economics, University of York, York, UK.; ^5^School of Economics, Peking University, Beijing, China.; ^6^School of Management, Beijing University of Chinese Medicine, Beijing, China.; ^7^Gabelli School of Business, Fordham University, New York City, NY, USA.

**Keywords:** World Management Survey, Hospital Management, Hospital Performance, Clinical Outcomes, Satisfaction, China

## Abstract

**Background:**

Improving healthcare productivity and efficiency through effective management practice is crucial in the healthcare sector. However, the evidence on how management practices affect hospital performance is mixed and limited in the public health system. The objectives of this study are (1) locating Chinese public hospitals’ management ability in the global health system community, and (2) investigating how public hospital’s management practice is correlated to the objective and subjective performances.

**Methods:**

Using the World Management Survey (WMS) methodology, the national Global Hospital Management SurveyChina (GHMS-China) was conducted from 2014 to 2016 to measure Chinese hospitals’ management practices. This study utilized a national representative hospital sample from the GHMS-China and used multi-variable linear regression model to examine the association between hospital performance and management practices. This study mainly focused on the clinical outcomes for acute myocardial infarction (AMI), heart failure (HF), pneumonia in children (PC), and coronary artery bypass grafting (CABG), as well as satisfaction measurements including staff turnover and subjective ratings from patient and staff.

**Results:**

Hospitals with higher management scores have significantly lower mortality rates on AMI, lower complication rates on CABG, and shorter average length of stay (LoS) for PC patients. Hospital management and subjective performance also shows a positive correlation, with a significant increase of inpatient satisfaction rating by 0.72 scores (95% CI: 0.28,1.16; *P*=.001). This relationship is more pronounced in hospitals with larger bed capacities, greater competition, more autonomy, and in sub-sample group of hospitals with superior management practice. The potential mechanisms through which hospital management can foster performance include attracting more talented clinical staffs, providing more valuable and continuous training opportunities, as well as providing more standardized clinical care service.

**Conclusion:**

Better management practice is correlated to superior hospital performance in Chinese Public Health Service System. Future studies with religious and causality study design are warranted.

## Background

**Key Messages**

**Implications for policy makers**

- The Global Hospital Management Survey-China (GHMS-China) based on the World Management Survey (WMS) methodology reveals that Chinese public hospitals’ overall management practice is not far behind the top performed country and even better than some developed countries such as France, Canada, and Italy. However, a significant disparity in management ability exists among Chinese public hospitals.
- Additional improvement is needed for retaining, managing, removing, and rewarding talent under the incentives management dimension; enhancing dialogue, consequence, and continuous improvement under the performance monitoring dimension; and better hospital layout design under the operations management dimension.
- Ensuring that public hospitals with good management practice operate in a competitive market with more autonomy is more likely for them to achieve a better performance.

**Implications for the public**

Improving hospital management practice is beneficial for patient’s well-being. Hospitals with a well-functioning staff management system to attract, promote, reward, and retain talented staffs are more likely to have an effective staff deploying procedure across departments, which is positively associated with a better healthcare experience for patients. Similarly, hospitals with better operations management capabilities are more likely to increase efficiency to reduce patient’s waiting time and improve patient’s feeling on care utilization. Moreover, hospitals with better target setting and management practices are more inclined to enhance the quality of clinical care, which is positively associated with patient’s improved clinical outcomes.

Over the past decades, there has been a notable increase in the focus on healthcare quality improvement.^1-3^ Nevertheless, the pace of enhancement in care quality has not met the expectations of many stakeholders,^4-8^ and considerable variability of care quality persists across different healthcare organizations.^9^ While substantial attention has been directed towards the implementation of evidence-based medicine—clinical practices that promote better care—there is a growing recognition of the importance of healthcare management practices that facilitate and motivate the delivery of high-quality care.^10-15^

 The investigation of high-performing healthcare facilities has been a focal point of interest in the field of healthcare management science for an extended period.^16^ The rationale is that providers are incentivized by their business objectives to enhance management practices to compete against diverse benchmarks, including price and quality.^17^ Substantial evidence has consistently supported this notion, indicating that management does matter for (private) providers from a range of perspectives: performance seems to be correlated with management practices, leadership, manager characteristics, and cultural attributes.^17-19^

 In the context of public healthcare sector, the involvement of governments is pivotal in shaping the configuration of management, establishing compensation structures for managers, and defining their responsibilities, rather than allowing healthcare enterprises to dictate these elements independently.^20^ As noted by Asaria et al,^20^ in such study settings, where management is perceived to be more an administrative than an entrepreneurial function, it is unclear how much management still matters for hospitals owned and oversighted by the government. This is true for either the English National Health Service or the Chinese Public Health Service System as well as other low- and middle-income countries. In such health systems hospitals have operated under governmental oversight aimed at compressing the number of managerial positions as well as capping their remuneration.^20^

 The existing evidence remains very limited and mixed,^20-24^ and it seems to be no correlation between management practices and public hospitals’ clinical performance.^20-23^ However, these studies did not utilize an internationally applicable tool for measuring management practices^20,22,23^ and were limited by the non-representative study sample^21^ as well as limited study focus, for example, mainly focusing on primary care facilities.^22,24^ Furthermore, as highlighted by Lega et al,^18^ there is a pressing need for further investigation to ascertain whether the lack of significant association between management and hospital performance is context-specific and to explore the potential generalization for other health system settings.

 To meet this knowledge gap, we implemented the Global Hospital Management Survey-China (GHMS-China) between 2014 and 2016,^25^ aimed at evaluating the management practices of hospitals in China. This survey was conducted in accordance with the internationally comparable World Management Survey (WMS) methodology.^19,26,27^ This framework facilitates a quantitative assessment and contextualization of the management performance of Chinese public hospitals within the broader global health system, including countries such as the United States, the United Kingdom, France, Germany, Italy, Canada, Sweden, Brazil, and India.^28^ Following this, we analyzed a nationally representative sample of public tertiary hospitals to examine the association between management practices and hospital performance, encompassing both objective clinical outcomes and subjective patient satisfaction metrics.

## Methods

###  The GHMS-China and the Main Independent Variable

 To measure the management practices of hospitals in China, we utilized the WMS methodology to launch the GHMS-China project from 2014 to 2016.^7^ By employing the Delphi method and hosting seminars for hospital administrators, research scholars, and policy-makers, we engaged in pre-survey interviews at 20 tertiary hospitals in China. Through considering respondents’ comprehension abilities and language habits, we tailored the survey instrument accordingly and developed the GHMS-China questionnaire. The questionnaire consists of 20 core question items in Supplementary file 1, divided into four dimensions: operations management (questions 1-4), performance monitoring (questions 5-9), targets management (questions 10-14), and incentives management (questions 15-20). More detailed information about the sampling, interviewing, and rating process is provided in Supplementary file 2.

 The raw overall management score is calculated by averaging the scores of the 20 management question items mentioned above. Each item has a scoring scale ranging from 1 to 5, with 1 represents worst practice, passive problem-solving and absence of institutionalized management and 5 represents best practice, proactive problem-solving, and institutionalized management with stringent adherence and safeguards. The range of hospital level overall management scores is also from 1 to 5.

 In the regression analysis below, we used the standardized overall management score rather than the raw overall management score, as suggested by the previous closely related literature.^28,29^ The standardized overall management score is calculated by standardizing the index to a mean of zero and a standard deviation of one. This is achieved by z-scoring the average of the z-scores obtained from the 20 individual management questions.^28^ Likewise, z-scores are calculated for the four management dimensions.

###  The Outcome Variables

 We utilized a variety of metrics to reflect hospital performance, including clinical outcomes and satisfaction ratings. Hospital performance indicators were provided by every hospital’s performance office during the survey period. To evaluate clinical care quality, we utilized risk-adjusted in-hospital mortality rates, average length of stay (LoS) for conditions like acute myocardial infarction (AMI), heart failure (HF), pneumonia in children (PC), and coronary artery bypass grafting (CABG) surgery, as well as complication rates specifically for CABG surgery. We extracted satisfaction ratings from outpatients, inpatients, and medical staff,^30^ and tracked the percentage of nurses leaving their jobs in the past year to gauge employee satisfaction.^29,31^

 The risk adjustment method is using hospital level Case-mix index to reflect the difference on patient and hospital characteristics among different hospitals, as suggested by and utilized in the previous literature.^32-35^ Performance indicators are limited to public tertiary hospitals. This study therefore examines the correlation between hospital performance and management practices based on data from 235 public tertiary hospitals.

###  Statistical Analysis

 We employ a multivariate linear regression model, considering that the performance variables, such as clinical outcomes and satisfaction ratings, are continuous variables. The formula utilized is as follows:


(1) $$y_{h}^{p}=\beta_{1}M_{rh}+\beta_{2}X_{h}+\beta_{3}X_{g}+\beta_{4}X_{n}+\mu_{rh}$$

Where $y_{h}^{p}$ represents the performance outcome *p* in hospital *h*, and all outcomes in the regression analysis are not standardized (not z-scored outcomes). ***M***_rh_ are the standardized management scores of the interviewee *r* in hospital *h*. We added the overall score, 4-dimensional score, and 20-practical score into the model separately. ***X***_h_ refers to the characteristics of the hospital *h*, including the duration year of hospital and its square term, the log value of hospital beds, hospital autonomy indicator (score 1-5, category variable), whether the hospital is managed by a third-party entity or part of a medical alliance, the percentage of managers receiving the clinical degrees or MBA degrees, and the number of hospital competitors (coded as 0 for none, 1 for less than five, and 2 for five or more, category variable). In addition, as for hospital geographical characteristics, denoted as ***X***_g_, we controlled city-level characteristics (population size, gross domestic product [GDP] per capita, the share of the primary industry in GDP, the share of the tertiary industry in GDP, and number of beds) and included province dummies.

***X***_n_ is a vector of management survey “noise” controls, including, (1) interviewee’s age, gender, education, department (cardiology, orthopedics or other), position (Director, Head Nurse or other), tenure of the interviewee, and interviewee’s proficiency in management practices (score 1-5, category variable); (2) the duration of the interview and the wave of the survey; and (3) the dummy of supervisor and interviewer. *u*_rh_ is the error term. Standard errors are clustered at the hospital level.

## Results

###  Descriptive Statistics

 The measurement of Chinese public hospitals’ management score and its distribution is shown in Figure. There is no hospital performing excellent (ie, overall management score is greater than 4) in management practice and only a few receiving an extremely low management rating (1 represents worst practice). The majority of hospitals obtain the management score ranging from 2 to 3.5, with a mean value of 2.74. Dimension and practice level management score is provided in Figure S1 and the international comparison of hospital management score is shown in Figure S2 (See Supplementary file 3). Figures S1 and S2 suggest that, although Chinese public hospitals perform weak on some specific practices/items under incentives management, performance monitoring, and operations management dimensions, the national level averaged overall management score is close to the best-performing country (ie, the US and the UK) and even better than some high-income countries, such as Canada and France.

**Figure:**
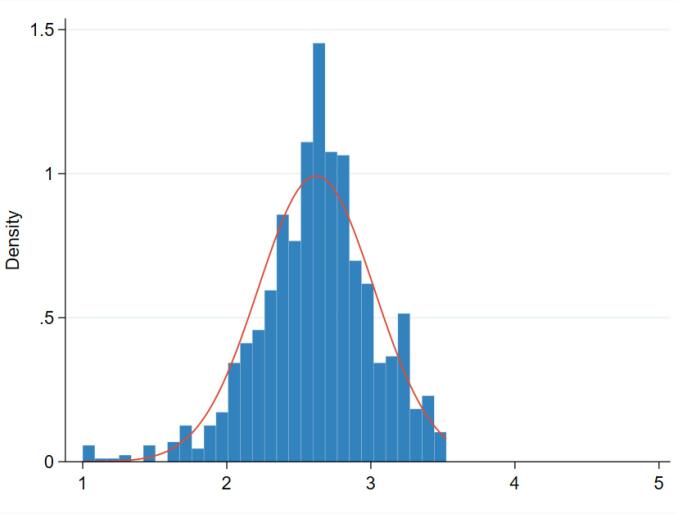
Hospital Management Scores in China. Notes: The data is from the GHMS-China. This figure shows the distribution of hospital management scores (1 = worst score, 5 = best score).

Table 1 presents more detailed descriptive statistics regarding management scores and performance outcomes from 235 public tertiary hospitals. Since all hospitals have around 2 interviews, the last column of Table 1 represents the total number of interviews (ie, the total number of hospitals times the number of interviews) instead of the total number of hospitals. The mean raw overall management score is 2.74 with a standard deviation of 0.42. Of which, sorting by the achieved average score, the best to worst performing sub-dimensions of management are operations management, targets setting, performance monitoring, and incentives management with the mean values of 2.86, 2.73, 2.70, and 2.70, respectively. The mean satisfaction of medical staff is 75.19, while the mean satisfaction of outpatients and inpatients are 82.76 and 90.18, respectively. The mean medical staff turnover is 3%. Additionally, the mean value of mortality rates for AMI, HF, PC, and CABG are 5.34%, 2.29%, 0.13%, and 2.06%, respectively. The mean LoS for CABG is 24.38 days, which is longer than AMI (9.06), HF (9.25), and PC (7.21). The mean complication rate for CABG is 3.83%. Table S1 includes descriptive statistics for hospital and geographic characteristics. The surveyed public tertiary hospitals exhibit an average building age of 70.78 years and an average bed capacity of 1809.30 beds. Around 83% of hospital managers hold clinical degrees, while only 7% possess Master of Business Administration (MBA) degrees. Furthermore, within a 30-minute travelling distance radius, 87.04% of hospitals face competition from at least one general hospital. The average population size of the surveyed cities stands at 728 210, with an average GDP per capita of 103 574.47 RMB (equivalent to around US$ 14 500). The descriptive statistics for noise controls is provided in Table S2 (See Supplementary file 3).

**Table 1:** Descriptive Statistics of Management Score and Performance Measures

**Variable**	**Mean**	**SD**	**Min.**	**Max.**	**N**
Management score (not z-scored)					
Overall management score	2.74	0.42	1.45	3.85	509
Operations management	2.86	0.41	1.50	4.00	509
Performance monitoring	2.70	0.48	1.20	4.00	509
Targets setting	2.73	0.53	1.00	4.00	509
Incentives management	2.70	0.51	1.17	4.17	509
Performance measures					
Satisfaction of outpatients	82.76	5.26	55.00	92.95	489
Satisfaction of inpatients	90.18	3.62	76.67	97.90	493
Satisfaction of medical staff	75.19	7.50	53.69	94.77	496
Staff turnover (nurses leaving in past 12 months)	0.03	0.05	0.00	0.33	509
Mortality from AMI (%)	5.34	4.45	0.00	32.41	455
LoS from AMI (day)	9.06	1.88	3.52	15.26	455
Mortality from HF (%)	2.29	2.16	0.00	12.87	455
LoS from HF (day)	9.25	1.79	4.62	16.73	455
Mortality from PC (%)	0.13	0.97	0.00	14.18	453
LoS from PC (day)	7.21	1.69	3.98	15.43	453
Mortality from CABG (%)	2.06	3.83	0.00	23.81	291
Complication from CABG (%)	3.83	9.56	0.00	60.72	291
LoS from CABG (day)	24.38	7.60	5.41	46.59	291

Abbreviations: SD, standard deviation; AMI, acute myocardial infarction; HF, heart failure; PC, pneumonia in children; CABG, coronary artery bypass grafting; LoS, length of stay. Notes: These are descriptive statistics of the dependent and independent variables. The data used in the analysis is 235 public tertiary hospitals from the GHMS-China from 2014 to 2016. It is important to note that the number of observations across different variables in this table varies slightly due to not all indicators being available for each hospital.

###  Regression Results

 In Table 2, the overall findings suggest that hospital management practice is positively associated with hospital performance. The columns (1) to (9) in Table 2 indicate that a one standard deviation increase in overall management score is significantly associated with a 0.56 (95% CI: -1.11, -0.01; *P* = .045) percentage point decrease in mortality rates for AMI, an reduction of 0.28 (95% CI: -0.48, -0.08; *P* = .006) days in average LoS for PC, and a 2.02 (95% CI: -4.01, -0.03; *P* = .046) percentage point decrease in complication rates for CABG. The subjective performance in column (11) also demonstrates that an increase in the overall management score is significantly correlated to a 0.72 (95% CI: 0.28, 1.16; *P* = .001) score increase in satisfaction ratings from inpatients.

**Table 2:** Hospital Performance and Overall Management Score

	**(1)**	**(2)**	**(3)**	**(4)**	**(5)**	**(6)**	**(7)**	**(8)**	**(9)**	**(10)**	**(11)**	**(12)**	**(13)**
	**AMI**		**HF**		**PC**		**CABG**			**Satisfaction Ratings and Turnover**			
Dependent variable	Mortality rates	LoS	Mortality rates	LoS	Mortality rates	LoS	Mortality rates	Complication rates	LoS	Outpatients	Inpatients	Medical staff	Staff turnover
Overall management store	-0.56* (0.28)	0.01 (0.13)	-0.14 (0.10)	-0.05 (0.12)	0.05 (0.05)	-0.28** (0.10)	-0.06 (0.25)	-2.02* (1.00)	-0.00 (0.62)	0.45 (0.34)	0.72** (0.22)	0.98 (0.51)	-0.00 (0.00)
R^2^	0.45	0.42	0.48	0.41	0.29	0.43	0.54	0.51	0.52	0.14	0.23	0.22	0.19
Observations	455	455	455	455	453	453	291	291	291	489	493	496	509
Mean values of the outcome	5.34	9.06	2.29	9.25	0.13	7.21	2.06	3.83	24.38	82.76	90.18	75.19	0.03

Abbreviations: AMI, acute myocardial infarction; HF, heart failure; PC, pneumonia in children; CABG, coronary artery bypass grafting; LoS, length of stay. Notes: The data is from the GHMS-China. ** and * represent significance at the 1% and 5% level, respectively. Standard errors are clustered at the hospital level. The number of observations in the table varies slightly because not all performance indicators are available for each hospital.

 Since overall management score can be divided into 4 sub-dimensions, including operations management, targets setting, performance monitoring, and incentives management, it gives us an opportunity to explore the connection between management and hospital performance in depth. In Table 3, the association between sub-management dimensions and hospital performance is presented in four panels. The Panel A suggest that hospitals with better operations management have a significant reduction in mortality rates for AMI and HF by 0.71 (95% CI: -1.24, -0.19; *P* = .008) and 0.23 (95% CI: -0.43, -0.03; *P* = .025) percentage points, respectively, a significant reduction in LoS for PC by 0.22 (95% CI: -0.38, -0.07; *P* = .005) inpatient days, and a significant improvement in inpatient satisfaction ratings by 0.52 (95% CI: 0.09, 0.95; *P* = .017) score. The Panel B indicates that enhanced performance monitoring is correlated to a 1.81 (95% CI: -3.18, -0.44; *P* = .010) percentage point decrease in complication rates for CABG and higher satisfaction ratings for inpatients (coefficient = 0.54, 95% CI: 0.14, 0.94; *P* = .008) and medical staff (coefficient = 0.97, 95% CI: 0.11, 1.82; *P* = .027). Panels C and D reveal that hospitals with better targets management and incentives management are associated with a significant LoS reduction by 0.21 (95% CI: -0.39, -0.04; *P* = .018) inpatient days for PC, and a significant improvement in satisfaction ratings for inpatients, with increases of 0.43 (95% CI: 0.06, 0.80; *P* = .025) and 0.51 (95% CI: 0.08, 0.93; *P* = .020) score, respectively. A further detailed analysis on the relationship between hospital practice level management ability and hospital performance is provided in Supplementary file 4.

**Table 3:** Hospital Performance and Four Dimensions of Management Scores

	**(1)**	**(2)**	**(3)**	**(4)**	**(5)**	**(6)**	**(7)**	**(8)**	**(9)**	**(10)**	**(11)**	**(12)**	**(13)**
	**AMI**		**HF**		**PC**		**CABG**			**Satisfaction Ratings and Turnover**			
Dependent variable	Mortality rates	LoS	Mortality rates	LoS	Mortality rates	LoS	Mortality rates	Complication rates	LoS	Outpatients	Inpatients	Medical staff	Staff turnover
Panel A: Operations management	-0.71** (0.27)	-0.05 (0.11)	-0.23* (0.10)	-0.15 (0.10)	0.02 (0.04)	-0.22** (0.08)	-0.01 (0.24)	-1.45 (0.98)	-0.18 (0.60)	0.50 (0.31)	0.52* (0.22)	0.45 (0.48)	-0.00 (0.00)
R^2^	0.45	0.42	0.49	0.42	0.29	0.43	0.54	0.50	0.52	0.14	0.22	0.22	0.19
Panel B: Performance monitoring	-0.26 (0.20)	-0.03 (0.11)	0.06 (0.12)	0.04 (0.11)	0.05 (0.05)	-0.15 (0.09)	0.17 (0.21)	-1.81** (0.69)	-0.20 (0.56)	0.30 (0.28)	0.54** (0.20)	0.97* (0.43)	-0.00 (0.00)
R^2^	0.44	0.42	0.48	0.41	0.29	0.43	0.54	0.51	0.52	0.14	0.22	0.23	0.18
Panel C: Targets management	-0.41 (0.26)	0.00 (0.12)	-0.15 (0.09)	-0.06 (0.11)	-0.01 (0.04)	-0.21* (0.09)	-0.08 (0.23)	-1.53 (0.81)	-0.05 (0.58)	0.09 (0.28)	0.43* (0.19)	0.82 (0.43)	-0.00 (0.00)
R^2^	0.44	0.42	0.48	0.41	0.29	0.43	0.54	0.50	0.52	0.14	0.22	0.22	0.19
Panel D: Incentives management	-0.24 (0.20)	0.10 (0.11)	-0.08 (0.10)	0.03 (0.10)	0.07 (0.06)	-0.21* (0.10)	-0.26 (0.27)	-0.95 (0.84)	0.40 (0.54)	0.38 (0.35)	0.51* (0.22)	0.43 (0.48)	0.00 (0.00)
R^2^	0.44	0.42	0.48	0.41	0.29	0.43	0.54	0.50	0.52	0.14	0.22	0.22	0.18
Observations	455	455	455	455	453	453	291	291	291	489	493	496	509
Mean values of the outcome	5.34	9.06	2.29	9.25	0.13	7.21	2.06	3.83	24.38	82.76	90.18	75.19	0.03

Abbreviations: AMI, acute myocardial infarction; HF, heart failure; PC, pneumonia in children; CABG, coronary artery bypass grafting; LoS, length of stay. Notes: The data is from the GHMS-China. ** and * represent significance at the 1% and 5% level, respectively. Standard errors are clustered at the hospital level.

###  Mechanism Analysis

 The benchmark regression analysis evidences a significant association between hospital performance and management score at overall, sub-dimensional, and specific practical levels. In this section, we delve deeper into exploring the potential mechanisms through which management practices influence hospital performance.

 Based on the previous literature, we identified three potential mechanisms through which better management practices could improve hospital performance, which include recruiting and maintaining high-quality healthcare professionals, providing continued learning/training opportunities, and promoting communication and understanding between patients and healthcare professionals. The positive association between these mechanisms and hospital performance both on objective (ie, mortality) and subjective (ie, satisfaction) indicators has been widely investigated and evidenced.^36-40^ Therefore, in this section, we focus on providing further evidence on the relationship between these mechanisms and hospital management practices.

 We proxied hospital’s ability to recruit and maintain high-quality healthcare professionals by the percentage of physicians with postgraduate degrees. For providing continued learning/training opportunities, we proxied it by using three specific indicators, including the number of physicians participating in the government organized skill trainings (log transformed), the number of physicians receiving continuing medical education (log transformed), and the number of physicians attending refresher course for more than six months (log transformed). For the relationship between patient and healthcare professionals, we used the number of medical disputes to proxy it. The result of mechanism analysis is presented in Table 4. The column (1) of Table 4 indicates that a one standard deviation increase in overall management score is significantly linked to a 1.01 (95% CI: 0.03, 1.98; *P* = .042) percentage point increase in the proportion of physicians holding postgraduate degrees. Performance monitoring (coefficient = 1.06, 95% CI: 0.30, 1.82; *P* = .007) and targets management (coefficient = 1.15, 95% CI: 0.22, 2.07; *P* = .015) dimensions both show a positive correlation with increased human capital on physicians.

**Table 4:** Mechanism Variables and Management Scores

	**(1)**	**(2)**	**(3)**	**(4)**	**(5)**
Dependent variable	Share of physicians with postgraduate degrees	#Physicians participating in the government organized skill trainings (log)	#Physicians receiving continuing medical education (log)	#Physicians attending refresher course for more than six months (log)	#Medical disputes (log)
Panel A: Overall management store	1.01* (0.49)	0.63* (0.29)	0.26* (0.10)	0.49** (0.14)	-0.04** (0.01)
R^2^	0.54	0.51	0.60	0.45	0.41
Panel B: Operations management	0.45 (0.43)	0.19 (0.24)	0.14 (0.08)	0.18 (0.11)	-0.03* (0.01)
R^2^	0.54	0.48	0.58	0.41	0.40
Panel C: Performance monitoring	1.06** (0.39)	0.45 (0.30)	0.12 (0.10)	0.33* (0.14)	-0.01 (0.01)
R^2^	0.55	0.49	0.57	0.43	0.40
Panel D: Targets management	1.15* (0.47)	0.47 (0.25)	0.26** (0.08)	0.51** (0.16)	-0.03** (0.01)
R^2^	0.55	0.50	0.60	0.46	0.41
Panel E: Incentives management	0.15 (0.42)	0.66* (0.29)	0.21* (0.10)	0.38** (0.13)	-0.03* (0.01)
R^2^	0.54	0.52	0.59	0.44	0.41
Observations	451	107	125	124	450
Mean values of the outcome	13.49	410.65	2597.47	45.28	0.41

Notes: The data is from the GHMS-China. ** and * represent significance at the 1% and 5% level, respectively. Standard errors are clustered at the hospital level.

 The columns (2) to (4) reveal that hospitals with higher management scores have more physicians engaged in training and continuing medical education. For instance, in Panel A, an overall management score is significantly related to a 63% increase (95% CI: 0.04, 1.22; *P* = .037) in physicians participating in government-organized skill training, a 26% increase (95% CI: 0.06, 0.46; *P* = .014) in physicians receiving continuing medical education, and a 49% increase (95% CI: 0.20, 0.78; *P* = .001) in physicians attending refresher course lasting more than six months.

 The column (5) suggests that hospitals with higher management scores experience fewer medical disputes. Panel A shows that hospitals implementing superior management practices have a 4% reduction (95% CI: -0.06, -0.01; *P* = .003) in medical disputes. Panels B to E indicate that enhanced operations management, targets management, and incentives management are both correlated to a significant reduction of 3% in medical disputes.

###  Heterogeneous Analysis

 In this section, we first divided the sample into two sub-groups based on the median values of hospital beds, competition levels, the percentage of managers receiving MBA degrees, and hospital autonomy status. The results are presented in Figure S3 (See Supplementary file 5) from Panels A to D. Panel A shows that hospitals with larger bed sizes have a stronger association between performance outcomes and overall management scores, such as lower mortality rates for AMI, shorter LoS for PC, and higher satisfaction from medical staff. Panel B indicates that hospitals facing more intensive competition have a more significant relationship on the association between overall management scores and lower mortality rates for AMI, as well as higher satisfaction among inpatients and medical staff. Panel C suggests that the percentage of managers receiving MBA degrees does not have a significant association with the outcomes. Panel D reveals that among hospitals with higher autonomy, a higher overall management score is associated with shorter LoS for PC and higher inpatient satisfaction.

 To explore how the relationship between hospital management and performance can be adjusted by hospital’s management ability, the overall management score was categorized into tertiles (lowest, middle, and highest management ability). The results in in Table S3 (See Supplementary file 5) indicate that the significant association between management and performance mainly appears in hospitals with the highest management ability (in third tertile). Specifically, for these hospitals with relative best management ability, a one standard deviation increase in overall management score is significantly associated with an increase of inpatient satisfaction by 0.98 score (95% CI: 0.08, 1.88; *P* = .033), a decrease of mortality rates in AMI and HF by 1.13 (95% CI: -2.00, -0.27; *P* = .011) and 0.64 (95% CI: -1.09, -0.20; *P* = .006) percentage points, respectively, a reduction of LoS for PC by 0.34 (95% CI: -0.63, -0.06; *P* = .019) inpatient days, and a decrease of CABG surgery complication rates by 6.68 (95% CI: -10.87, -2.49; *P* = .002) percentage point.

## Discussion

 The importance of management on hospital performance in government-owned and highly centralized healthcare systems is rarely known. Using the GHMS-China data from 2014 to 2016, this study aims to fill this knowledge gap by measuring hospital management practices in details (at overall, dimension, and specific practice levels) and examining their relationship with subjective and objective performance. The results show that good management is significantly correlated with better clinical outcomes and higher satisfaction ratings. Specifically, hospitals with higher overall management scores have significantly lower mortality rates for AMI, shorter average LoS for PC, and lower complication rates for CABG. Those high-performing hospitals also receive better satisfaction ratings from inpatients. This relationship is more pronounced in hospitals with larger bed sizes, more competitive capacity, and greater autonomy, as well as in sub-sample group of hospitals with superior management practice (in third tertile).

 The findings of this study align with previous literature emphasizing the crucial role of management to organizational performance. Previous literature has consistently shown that effective management practices lead to positive performance outcomes in manufacturing^41-44^ and education sectors.^45,46^ In healthcare sector, there is a growing body of literature trying to explore the relationship between hospital management and performance, and the existing evidence suggests that standard management practices might be able to enhance operations and, to some extent, improve quality of care.^28,29,47-51^ However, the existing evidence is not only highly mixed on some narrow but commonly used objective performance measures (ie, re-admission rate, LoS and mortality) but also lack subjective patient centered measurements such as satisfaction ratings. More importantly, to the best of our knowledge, almost all research was conducted in the high-income countries where the healthcare system is driven by consumerism and customer choice. In such a case, the policy implications under this topic might be unsuitable for some of other healthcare systems where the planning, budgeting, auditing, and governing instead of consuming play a critical role in driving high-quality care provision. In this study, our results show that hospital’s overall, four dimensional, and twenty practical management scores are either associated with better objective care quality indicators or beneficial for them to get a higher inpatient subjective rating score in a government owned and centralized health system.

 This study also fills the existing knowledge gap by revealing the potential mechanisms through which hospital management can improve performance, which is rarely known by previous literature. This study provides correlational evidence on the positive relationship between hospital management and staff recruiting, training and medical dispute avoiding (proxy the standardization of clinical care operations). It suggests that the potential main method for hospitals to reach a higher level of performance is (1) setting good management on performance monitoring and targeting to attract more talented clinical staffs; (2) establishing good target tracking and incentivizing program to drive clinical staffs continuously receive high-quality training; and then (3) improving the standardization of clinical care service operation and delivery due to those talented clinical staffs have been attracted, trained and maintained. From this perspective, considering the place (China) where this study was done, better hospital performance is not always driven by consumer choice as previously suggested by other studies.

 The implication of this study is that, as a developing country, China has a strong position in the global hospital management community. As suggested by the descriptive results of the GHMS-China, the management practice (the overall score in Figure S2) of Chinese hospitals is not far away from the top performed country (the United States) and it is close to many developed countries such as the United Kingdom, Sweden, Germany, and Canada. Moreover, as shown in Figure S1, Chinese hospitals perform very well in standardization and protocols, target balance, attracting talent and rewarding high performers. However, Chinese hospitals have more space to improve their management practice in incentives management, performance monitoring, and operations management dimensions. This is the potential fields that policy-makers should input more resources in the future.

###  Limitations of the Study

 This study has some limitations. First, our use of cross-sectional data makes it difficult to establish a causal relationship between management and performance. Second, our analysis is restricted to performance indicators for public tertiary hospitals in China, which may limit the generalizability of our findings. Third, the data used for the analysis is from 2014 to 2016 and is not the most recent. Nonetheless, there have been no major institutional changes in Chinese health system and regulations since we collected this dataset. Therefore, we believe that the age of the data may not significantly impact the conclusions and inferences drawn.

## Conclusion

 Based on the GHMS-China data from 2014 to 2016, we examine the correlation between hospital performance and management practices. The findings indicate that hospitals that implement more effective management practices tend to achieve superior clinical outcomes and higher satisfaction ratings among inpatients. The heterogeneity analysis results show that the correlations mentioned above are more pronounced in hospitals with a larger number of hospital beds, greater competition, and more autonomy, as well as in sub-sample group of hospitals with superior management practice (in third tertile). The potential mechanisms through which management practice could improve hospital performance are recruiting talented clinical staff, providing continuous learning and training opportunities, and improving the standardization of clinical care services.

## Acknowledgements

 We acknowledge the support by the National Natural Science Foundation of China. We also thank all participants and data investigators for the GHMS-China.

## Ethical issues

 The survey in this study was for healthcare institutions and ethical permission from appropriate bodies was not required.

## Conflicts of interest

 Authors declare that they have no conflicts of interest.

## Data availability statement

 The datasets analyzed during the current study are available from the corresponding author on reasonable request.

## Supplementary files


Supplementary file 1. List of Management Practices.

Supplementary file 2. The Sampling, Interviewing, and Rating Process of GHMS-China.

Supplementary file 3. Descriptive Analysis‎.

Supplementary file 4. Further Analysis.

Supplementary file 5. Heterogeneous Analysis.
