# Supplementary material for: How Does Management Matter for Hospital Performance? Evidence From the Global Hospital Management Survey in China
Source: Int J Health Policy Manag. 2024 Dec 9;13:8478. doi: 10.34172/ijhpm.8478 (PMC11806224; doi:10.34172/ijhpm.8478)
Supplement: Supplementary file 1 — List of Management Practices. [file ijhpm-13-8478-s001.pdf]

Article title: How Does Management Matter for Hospital Performance? Evidence From the Global Hospital Management Survey in China

Journal name: International Journal of Health Policy and Management (IJHPM)

Authors' information: Qinghong He[1]¶, Gordon G. Liu[2,3]*, Jinyang Chen[4]¶, Luoqi Yuan[5], Xuezhi Hong[6], Zhihua Zhang[7]

[1]Institute of Economics, Chinese Academy of Social Sciences, Beijing, China.

[2]Institute for Global Health and Development, National School of Development, Peking University, Beijing, China.

[3]China Center for Health Economic Research (CCHER), Peking University, Beijing, China.

[4]Centre for Health Economics, University of York, York, UK.

[5]School of Economics, Peking University, Beijing, China.

[6]School of Management, Beijing University of Chinese Medicine, Beijing, China.

[7]Gabelli School of Business, Fordham University, New York City, NY, USA.

¶Both authors contributed equally to this paper.

***Correspondence to:** Gordon G. Liu; Email: gordonliu@nsd.pku.edu.cn

Citation: He Q, Liu GG, Chen J, Yuan L, Hong X, Zhang Z. How does management matter for hospital performance? Evidence from the global hospital management survey in China. Int J Health Policy Manag. 2024;13:8478. doi:10.34172/ijhpm.8478

Supplementary file 1. List of Management Practices

Management practices	Abbreviations
**Operations Management**	
Q1. Layout of Patient Flow	O1
Q2. Rationale for Introducing Standardization/Pathway Management	O2
Q3. Standardization and Protocols	O3
Q4. Good Use of Human Resources	O4

**Performance Monitoring**	
Q5. Continuous Improvement	M1
Q6. Performance Tracking	M2
Q7. Performance Review	M3
Q8. Performance Dialogue	M4
Q9. Consequence Management	M5
**Targets Management**	
Q10. Target Balance	T1
Q11. Target Interconnection	T2
Q12. Time Horizon of Targets	T3
Q13. Target Stretch	T4
Q14. Clarity and Comparability of Targets	T5
**Incentives management**	
Q15. Rewarding High Performers	I1
Q16. Fixing Poor Performers	I2
Q17. Promoting High Performers	I3
Q18. Managing Talent	I4
Q19. Retaining Talent	I5
Q20. Creating a distinctive employee value proposition to attract talent	I6
