# Supplementary material for: How Does Management Matter for Hospital Performance? Evidence From the Global Hospital Management Survey in China
Source: Int J Health Policy Manag. 2024 Dec 9;13:8478. doi: 10.34172/ijhpm.8478 (PMC11806224; doi:10.34172/ijhpm.8478)
Supplement: Supplementary file 2 — The Sampling, Interviewing, and Rating Process of GHMS-China. [file ijhpm-13-8478-s002.pdf]

Article title: How Does Management Matter for Hospital Performance? Evidence From the Global Hospital Management Survey in China

Journal name: International Journal of Health Policy and Management (IJHPM)

Authors' information: Qinghong He[1]¶, Gordon G. Liu[2,3]*, Jinyang Chen[4]¶, Luoqi Yuan[5], Xuezhi Hong[6], Zhihua Zhang[7]

[1]Institute of Economics, Chinese Academy of Social Sciences, Beijing, China.

[2]Institute for Global Health and Development, National School of Development, Peking University, Beijing, China.

[3]China Center for Health Economic Research (CCHER), Peking University, Beijing, China.

[4]Centre for Health Economics, University of York, York, UK.

[5]School of Economics, Peking University, Beijing, China.

[6]School of Management, Beijing University of Chinese Medicine, Beijing, China.

[7]Gabelli School of Business, Fordham University, New York City, NY, USA.

¶Both authors contributed equally to this paper.

***Correspondence to:** Gordon G. Liu; Email: gordonliu@nsd.pku.edu.cn

Citation: He Q, Liu GG, Chen J, Yuan L, Hong X, Zhang Z. How does management matter for hospital performance? Evidence from the global hospital management survey in China. Int J Health Policy Manag. 2024;13:8478. doi:10.34172/ijhpm.8478

Supplementary file 2. The Sampling, Interviewing, and Rating Process of GHMS-China

The eligible hospitals for the survey GHMS-China are (i) general hospitals; and (ii) with cardiology or orthopedics departments. These departments are selected due to their larger size and extensive interconnections with other hospital departments, and interviews were conducted with the Director and Head Nurse of the departments. Additionally, a small number of Medical Services Section and Nursing department directors were included to validate responses and prevent bias from a single type of interviewee. We collaborated with esteemed institutions such as Peking University, Tsinghua University, the China Hospital Association, and the Cardiovascular Branch of the Chinese Medical Association to make sure that we could have enough hospitals as the interview sample.

The survey was completed with 510 hospitals, situated in 31 provinces throughout the country. Of which, 265 were tertiary hospitals and 245 were secondary hospitals; 482 hospitals were publicly owned, while 28 were privately owned. The response rate stands at 18.6%, which is similar to the 21% response rate observed among eligible hospitals in the United States[28]. The low response rate is predominantly attributed to incomplete interviews by some hospital interviewees, resulting in their exclusion from our analysis sample.

To elicit candid responses from the interviewee to capture the true level of management practices in the natural state of the hospitals, we initially used a scientific double-blind interview method.[9] The interview was carried out via telephone without disclosing to the interviewees being scored. Interviewees were also unaware of the interviewer's identity, background, and questionnaire details. The purpose of the interviews, as communicated to the interviewees, is to gain an accurate understanding of the management practices at their hospital, ensuring genuine responses. Interviewers were not briefed about the hospital or the interviewees beforehand, receiving only the hospital's code number and the interviewee's phone number. This approach ensures that the scoring is based on the interviewer's assessment of the actual practices at the hospital, rather than on idealized perceptions, participant perceptions, or interviewer biases.

We recruited outstanding college students with a background in economics, sociology, or management as interviewers. To achieve consistency in interview procedures and scoring standards across the project team and to enable international comparability, all interviewers received a week of unified training. We worked closely with the WMS international team to make sure that the Chinese training materials was in line with international standards. During the interviews, each interviewee was asked 3-4 open-ended questions by 2 interviewers (1 to ask questions and 1 to record key points) about each management practice to avoid the possibility that interviewees may tend to choose positive answers when faced with closed-ended questions, which may result in high interview scores. For example, "*what are the goals of your hospital or your department?*" required interviewees to provide specific examples, which helped us to verify the accuracy of their answers. To maintain quality, we randomly selected 10% of interviewees for supervision.

Two interviewers independently scored each management practice based on interviewee answers, guided by globally standardized scoring criteria. The scores were then reviewed, discussed, and harmonized, particularly for practices with inconsistent scores. In cases involving supervisors, they were also engaged in the discussion and received harmonized scores. Scores for each management practice were determined based on the standardization of the management system, level of proactivity, and degree of staff participation.
