# Supplementary material for: How Does Management Matter for Hospital Performance? Evidence From the Global Hospital Management Survey in China
Source: Int J Health Policy Manag. 2024 Dec 9;13:8478. doi: 10.34172/ijhpm.8478 (PMC11806224; doi:10.34172/ijhpm.8478)
Supplement: Supplementary file 3 — Descriptive Analysis‎. [file ijhpm-13-8478-s003.pdf]

Article title: How Does Management Matter for Hospital Performance? Evidence From the Global Hospital Management Survey in China

Journal name: International Journal of Health Policy and Management (IJHPM)

Authors' information: Qinghong He[1]¶, Gordon G. Liu[2,3]*, Jinyang Chen[4]¶, Luoqi Yuan[5], Xuezhi Hong[6], Zhihua Zhang[7]

[1]Institute of Economics, Chinese Academy of Social Sciences, Beijing, China.

[2]Institute for Global Health and Development, National School of Development, Peking University, Beijing, China.

[3]China Center for Health Economic Research (CCHER), Peking University, Beijing, China.

[4]Centre for Health Economics, University of York, York, UK.

[5]School of Economics, Peking University, Beijing, China.

[6]School of Management, Beijing University of Chinese Medicine, Beijing, China.

[7]Gabelli School of Business, Fordham University, New York City, NY, USA.

¶Both authors contributed equally to this paper.

***Correspondence to:** Gordon G. Liu; Email: gordonliu@nsd.pku.edu.cn

Citation: He Q, Liu GG, Chen J, Yuan L, Hong X, Zhang Z. How does management matter for hospital performance? Evidence from the global hospital management survey in China. Int J Health Policy Manag. 2024;13:8478. doi:10.34172/ijhpm.8478

Supplementary file 3. Descriptive Analysis

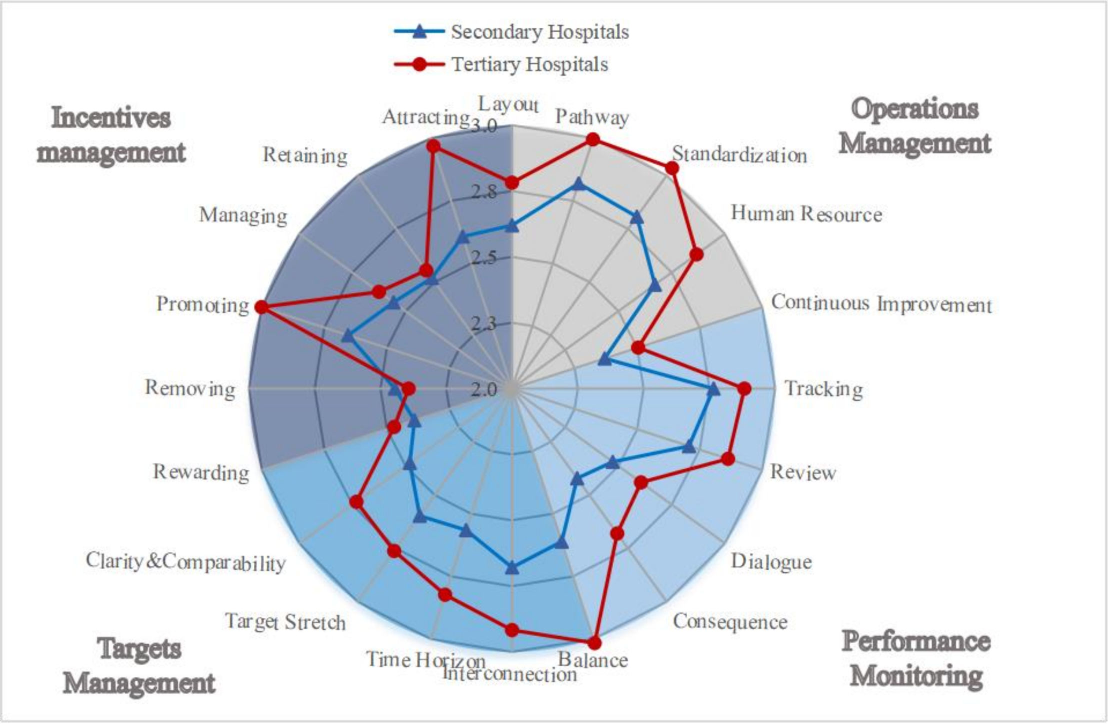

Figure S1. Radar Chart of Twenty Management Practice Scores

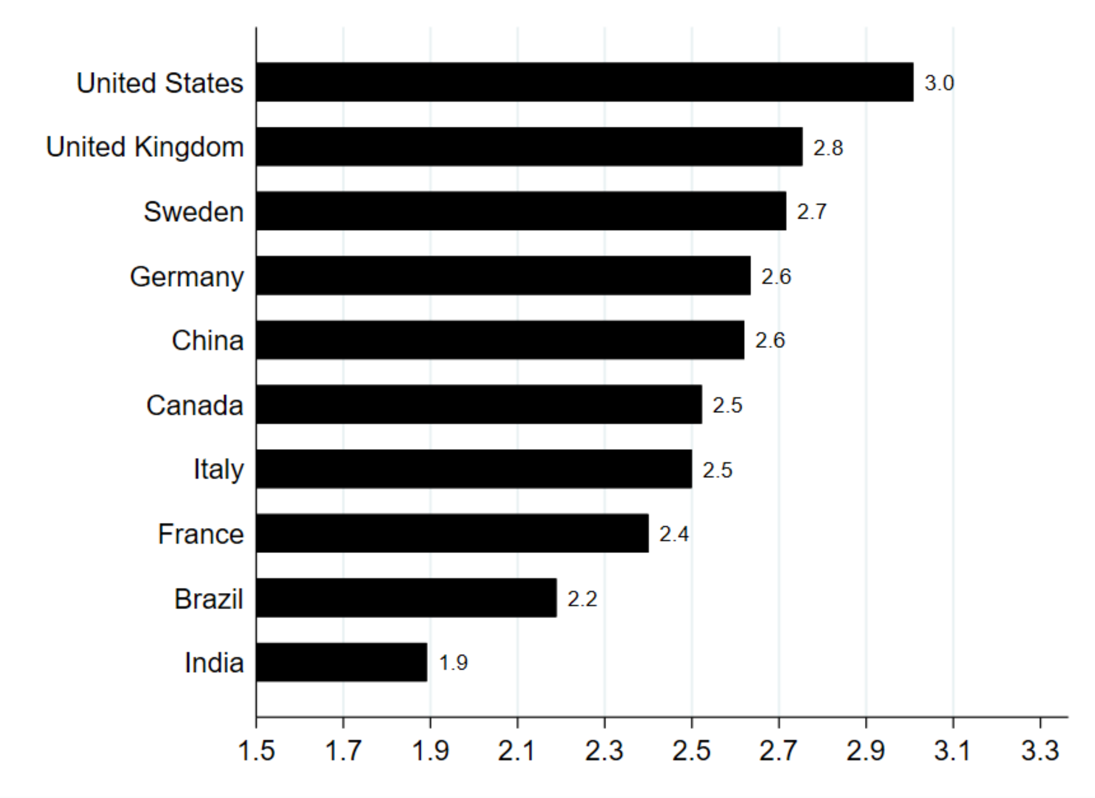

Figure S2. Hospital Management Scores across Countries

Notes: The figure illustrates the average management score on a scale of 1 to 5 across different countries. The average score is calculated by averaging the responses to twenty individual questions within each hospital, and then taking the unweighted average across all hospitals within each country. The dark bar represents the average score without any control. The data for all countries, except China, is sourced from Bloom et al (2020)[28], while the hospital management data for China is obtained from GHMS-China. The number of hospital observations for each country are as follows: Brazil (286), from Canada (174), China (510), France (147), Germany (124), India (490), Italy (154), Sweden (43), United Kingdom (235), and United States (307).

Table S1. Descriptive Statistics of Hospital and Geographic Characteristics

Variable	Mean	SD	Min	Max	N
Hospital characteristics					
Building age (years)	70.78	27.86	1	154	509
Number of beds	1809.30	1113.78	0	6750	509
The percentage of managers receiving clinical degrees (%)	82.92	24.25	0	100	509
The percentage of managers receiving MBA degrees (%)	6.60	17.02	0	100	509
Whether the hospital is managed by a third-party company	No. (%)				
No	498 (98.42%)		0	1	509
Yes	8 (1.58%)		0	1	509
Whether the hospital is part of a medical alliance	No. (%)				
No	471 (92.90%)		0	1	509
Yes	36 (7.10%)		0	1	509
Number of competitors	No. (%)				
0 for none	66 (12.97%)		0	1	509
1 for less than five	318 (62.48%)		0	1	509
2 for five or more	125 (24.56%)		0	1	509
Hospital autonomy	No. (%)				
1 (Very limited autonomy)	162 (31.83%)		0	1	509
2 (Lower than half autonomy)	14 (2.75%)		0	1	509
3 (Half autonomy)	309 (60.71%)		0	1	509
4 (More than half autonomy)	3 (0.59%)		0	1	509
5 (Full autonomy)	13 (2.55%)		0	1	509
Missing	8 (1.57%)		0	1	509
Geographic characteristics					
Population size (thousand)	728.21	523.10	10.51	3392	509

Gross domestic product per capita (RMB)	103574.47	86995.32	18437	506301	509
Share of the primary industry in GDP (%)	6.24	5.60	.04	25.28	509
Share of the tertiary industry in GDP (%)	51.73	13.56	27.09	80.23	509
Number of beds	47727.42	35165.66	1178	177410	509

Notes: These are descriptive statistics of hospital and geographic characteristics variables. The data used in the analysis is 235 public tertiary hospitals from the GHMS-China from 2014 to 2016.

Abbreviations: AMI, Acute Myocardial Infarction; HF, Heart Failure; PC, Pneumonia in Children; CABG, Coronary Artery Bypass Grafting, LoS, Length of stay in the hospital.

Table S2. Descriptive Statistics of Noise Controls

**Characteristics**	**Categorical Variables, No. (%)**
Gender of the interviewee	
Female	373 (73.28%)
Male	136 (26.72%)
Education of the interviewee	
Technical secondary school	4 (0.79%)
Junior college	27 (5.30%)
Bachelor's degree	327 (64.24%)
Master degree	102 (20.04%)
Doctor degree	49 (9.63%)
Position of the interviewee	
Director of the department	185 (36.35%)
Deputy director of the department	12 (2.36%)
Head Nurse of the department	287 (56.39%)
Director of Nursing department	11 (2.16%)
Nurse	14 (2.75%)
Department of the interviewee	
Cardiology department	222 (43.61%)
Orthopedic department	227 (44.60%)
Nursing department	30 (5.89%)
Medical Services Section	28 (5.50%)
Other	2 (0.39%)
Interviewee's proficiency in management practices	
1 (Have limited knowledge of their department, but nothing else about the hospital)	2 (0.39%)

2 (Lower than full knowledge and more than limited knowledge of their department)	8 (1.57%)
3 (Full knowledge of their department, but limited knowledge of the rest of the hospital)	112 (22.00%)
4 (Lower than full knowledge and more than limited knowledge about the rest of the hospital)	268 (52.65%)
5 (Full knowledge about their department and the rest of the hospital)	119 (23.38%)
Wave of the survey	
Wave 1	165 (32.42%)
Wave 2	100 (19.65%)
Wave 3	167 (32.81%)
Wave 4	77 (15.13%)
	**Continuous Variables, Mean (SD)**
Age of the interviewee (years)	44.00 (6.16)
Tenure of the interviewee (years)	8.33 (5.62)
Duration of the interview (minutes)	43.86 (13.74)
Number of the interviewee in each hospital	2.26 (1.00)

Notes: These are descriptive statistics of the noise controls variables. The data used in the analysis is 235 public tertiary hospitals from the GHMS-China from 2014 to 2016.
