# Supplementary material for: How Does Management Matter for Hospital Performance? Evidence From the Global Hospital Management Survey in China
Source: Int J Health Policy Manag. 2024 Dec 9;13:8478. doi: 10.34172/ijhpm.8478 (PMC11806224; doi:10.34172/ijhpm.8478)
Supplement: Supplementary file 4 — Further Analysis. [file ijhpm-13-8478-s004.pdf]

Article title: How Does Management Matter for Hospital Performance? Evidence From the Global Hospital Management Survey in China

Journal name: International Journal of Health Policy and Management (IJHPM)

Authors' information: Qinghong He[1]¶, Gordon G. Liu[2,3]*, Jinyang Chen[4]¶, Luoqi Yuan[5], Xuezhi Hong[6], Zhihua Zhang[7]

[1]Institute of Economics, Chinese Academy of Social Sciences, Beijing, China.

[2]Institute for Global Health and Development, National School of Development, Peking University, Beijing, China.

[3]China Center for Health Economic Research (CCHER), Peking University, Beijing, China.

[4]Centre for Health Economics, University of York, York, UK.

[5]School of Economics, Peking University, Beijing, China.

[6]School of Management, Beijing University of Chinese Medicine, Beijing, China.

[7]Gabelli School of Business, Fordham University, New York City, NY, USA.

¶Both authors contributed equally to this paper.

***Correspondence to:** Gordon G. Liu; Email: gordonliu@nsd.pku.edu.cn

Citation: He Q, Liu GG, Chen J, Yuan L, Hong X, Zhang Z. How does management matter for hospital performance? Evidence from the global hospital management survey in China. Int J Health Policy Manag. 2024;13:8478. doi:10.34172/ijhpm.8478

Supplementary file 4. Further Analysis

Since the GHMS-China survey consists of twenty management practices' questions under the four sub-management dimensions mentioned above, we further investigated how specific 20 management practices correlated to hospital performance.

Panels A to E in Figure A1 show that hospitals that implement standardization (O2) and balanced targets (T1) tend to have lower mortality rates for conditions like AMI and HF, as well as shorter LoS for patients with HF and PC. Additionally, Panel F suggests that fixing poor performers and rewarding high performers (I1, I2) is linked to reduced LoS for PC. Panel I indicates that hospitals implementing practices such as reviewing, dialogue, and continuous performance improvement (M1, M3, M4) have lower complication rates for procedures like CABG.

Furthermore, Panels A to D in Figure A2 examine the relationship between satisfaction ratings and the twenty sub-management practices. Panel A shows that hospitals that implement standardized procedures and protocols (O3), set appropriate time targets (T3), and retain talent (I5) tend to have higher satisfaction ratings from outpatients. Panel B reveals that hospitals introducing standardized procedures and protocols (O3), reviewing and continuous improvement performance (M1, M3), setting interconnected and comparable targets (T2, T5), and fixing poor performers (I2) have higher satisfaction ratings from inpatients. Panel C demonstrates that hospitals implementing standardization (O2), performance dialogue (M4), and target interconnection (T2) receive higher satisfaction ratings from medical staff. Panel D indicates that the clarity and comparability of targets (T5) is associated with lower staff turnover.

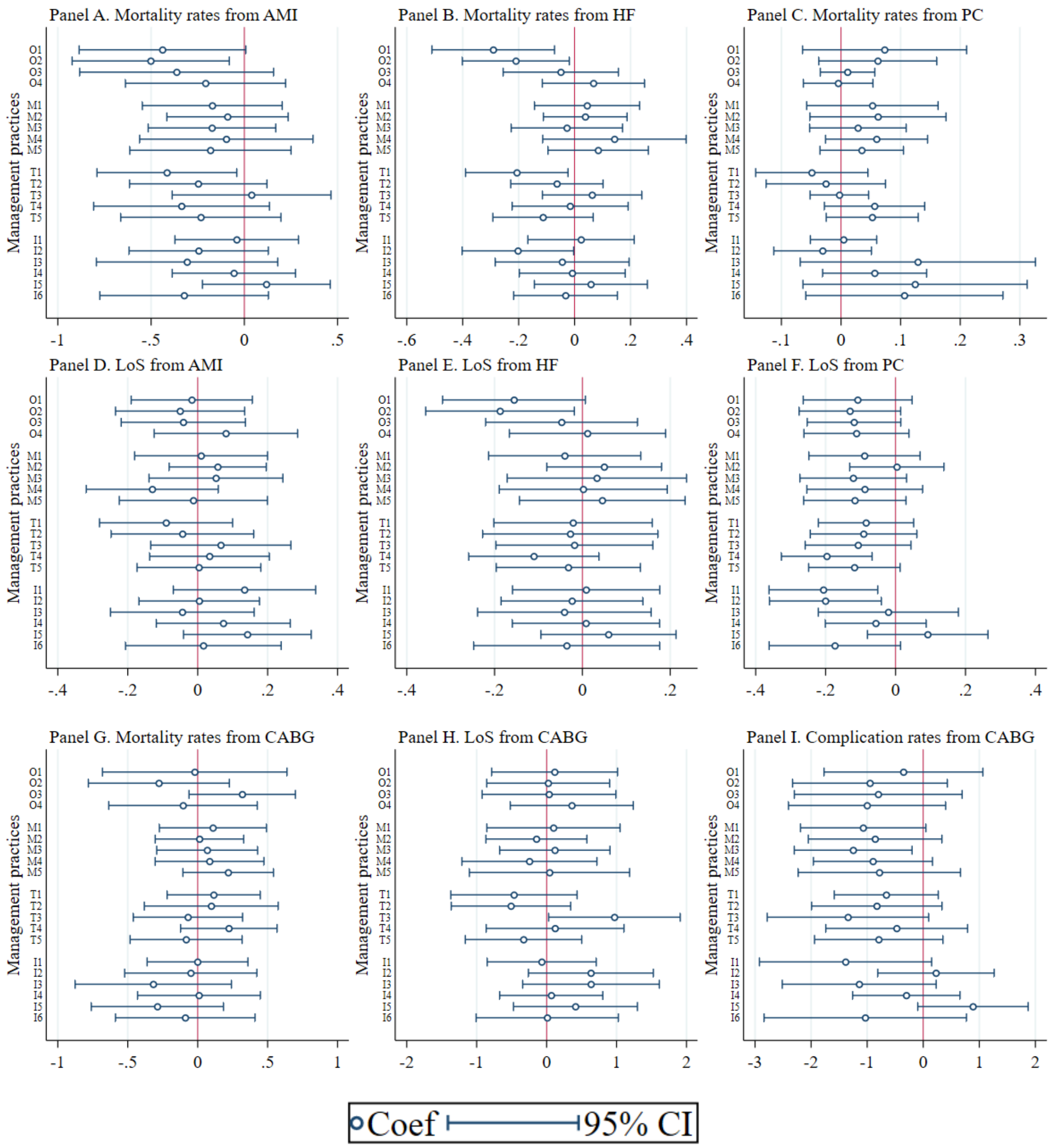

Figure A1. The Correlation between Clinical Outcomes and Twenty Sub-Management Practices

Notes: The data is from the GHMS-China. In the graph, the plotted points represent regression coefficients, and the blue lines show 95% confidence intervals determined using equation (1) with standard errors clustered at the hospital level. Panel A to Panel F uses the mortality rates and LOS from AMI, HF, and PC, while Panel G to Panel I employ mortality rates, LoS, and complication rates from CABG as dependent variables. In all panels, operations management are coded as the questions O1-O4, performance monitoring as M1-M5, targets management as T1-T5, and incentives management as I1-I6 (**Supplementary file 1**).

Abbreviations: AMI, Acute Myocardial Infarction; HF, Heart Failure; PC, Pneumonia in Children; CABG, Coronary Artery Bypass Grafting; LoS, length of stay in the hospital.

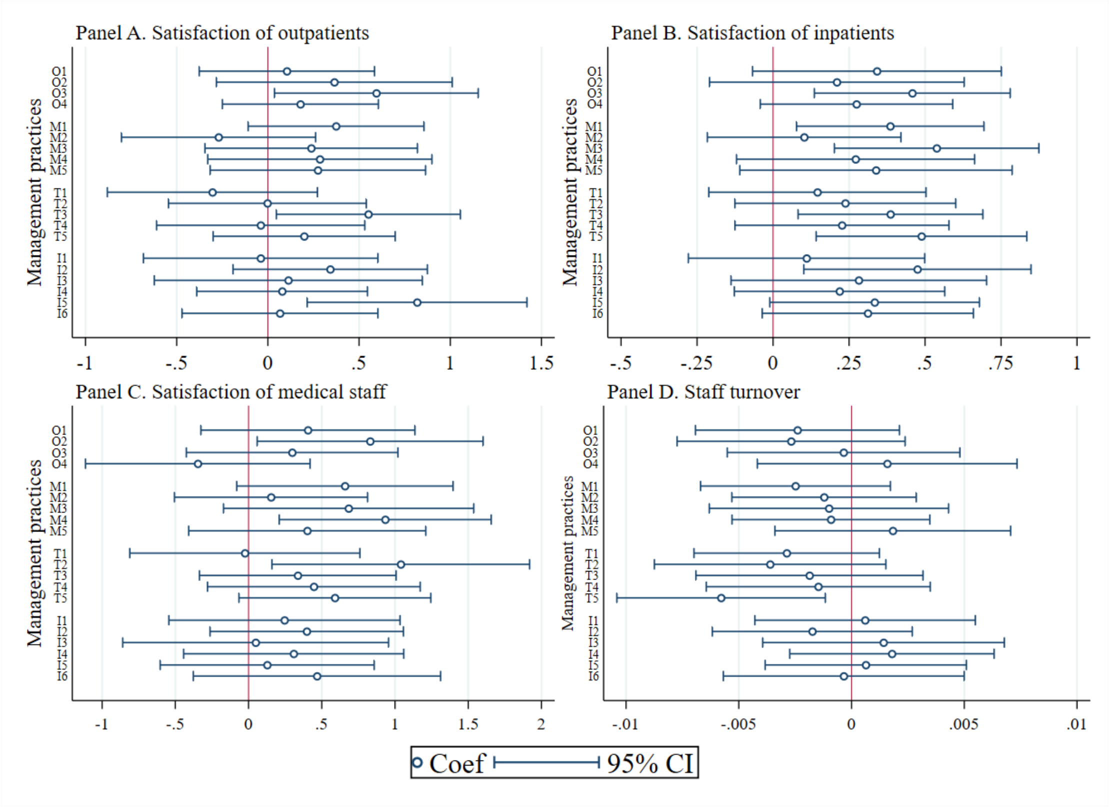

Figure A2. The Correlation between Satisfaction Ratings and Twenty Sub-Management Practices

Notes: The data is from the GHMS-China. In the graph, the plotted points represent regression coefficients, and the blue lines show 95% confidence intervals determined using equation (1) with standard errors clustered at the hospital level. Panel A to Panel C uses the satisfaction of outpatients, inpatients, and medical staff, while Panel D employ staff turnover as dependent variables. In all panels, operations management are coded as the questions O1-O4, performance monitoring as M1-M5, targets management as T1-T5, and incentives management as I1-I6 (**Supplementary file 1**).
