# Supplementary material for: How Does Management Matter for Hospital Performance? Evidence From the Global Hospital Management Survey in China
Source: Int J Health Policy Manag. 2024 Dec 9;13:8478. doi: 10.34172/ijhpm.8478 (PMC11806224; doi:10.34172/ijhpm.8478)
Supplement: Supplementary file 5 — Heterogeneous Analysis. [file ijhpm-13-8478-s005.pdf]

Article title: How Does Management Matter for Hospital Performance? Evidence From the Global Hospital Management Survey in China

Journal name: International Journal of Health Policy and Management (IJHPM)

Authors' information: Qinghong He[1]¶, Gordon G. Liu[2,3]*, Jinyang Chen[4]¶, Luoqi Yuan[5], Xuezhi Hong[6], Zhihua Zhang[7]

[1]Institute of Economics, Chinese Academy of Social Sciences, Beijing, China.

[2]Institute for Global Health and Development, National School of Development, Peking University, Beijing, China.

[3]China Center for Health Economic Research (CCHER), Peking University, Beijing, China.

[4]Centre for Health Economics, University of York, York, UK.

[5]School of Economics, Peking University, Beijing, China.

[6]School of Management, Beijing University of Chinese Medicine, Beijing, China.

[7]Gabelli School of Business, Fordham University, New York City, NY, USA.

¶Both authors contributed equally to this paper.

***Correspondence to:** Gordon G. Liu; Email: gordonliu@nsd.pku.edu.cn

Citation: He Q, Liu GG, Chen J, Yuan L, Hong X, Zhang Z. How does management matter for hospital performance? Evidence from the global hospital management survey in China. Int J Health Policy Manag. 2024;13:8478. doi:10.34172/ijhpm.8478

Supplementary file 5. Heterogeneous Analysis

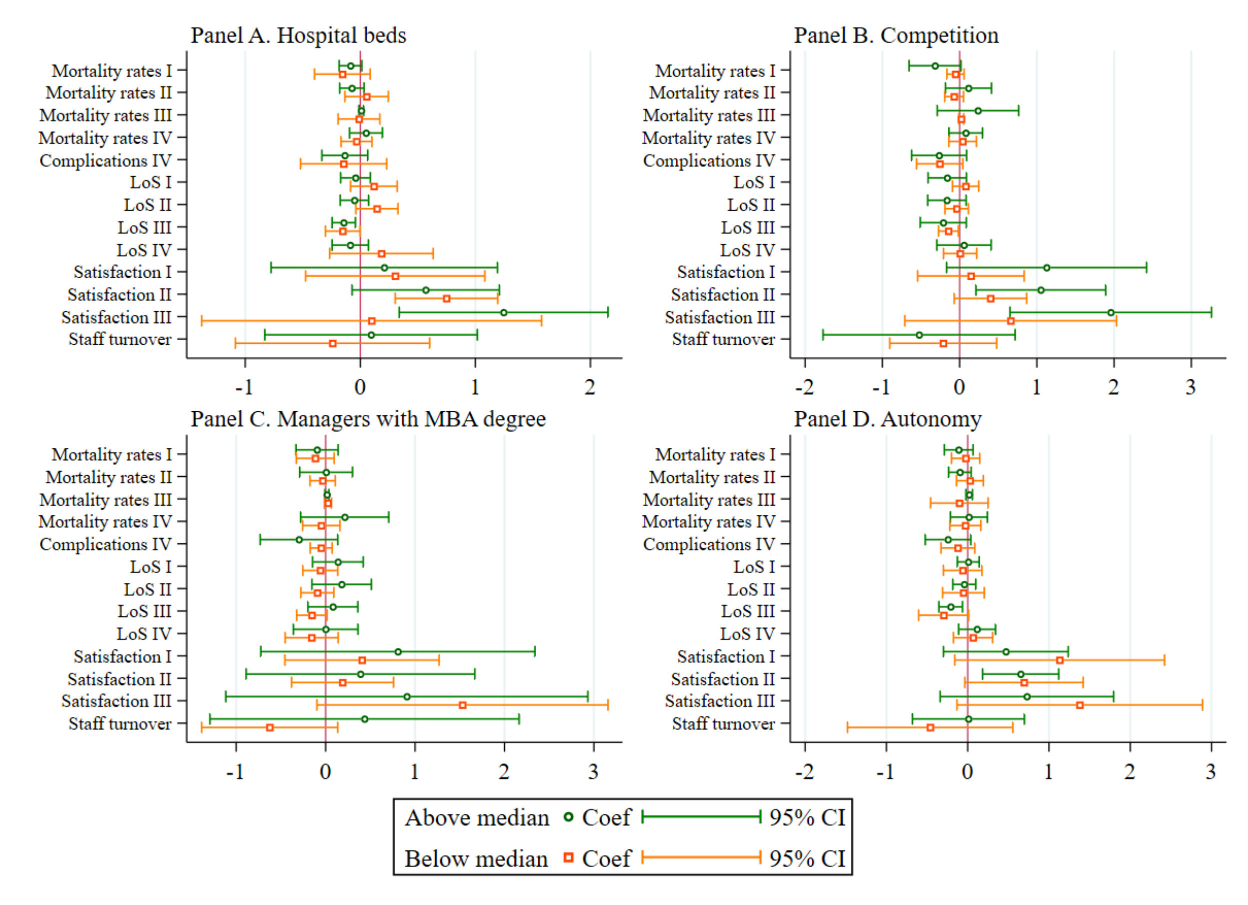

Figure S3. Heterogeneous Analysis between Hospital Performance and Overall Management Score

Notes: The data is from the GHMS-China. The figure from Panel A to Panel D divides the sample by the median values of hospital beds, competition levels, the percentage of managers receiving MBA degrees and autonomy. In the graph, the plotted points represent regression coefficients, and the blue lines show 95% confidence intervals from separate regression estimations of equation (1) for the sub-sample. In all panels, we use the z-score of the overall management score. Additionally, the regression coefficients and 95% confidence intervals for staff turnover are minimal. To visually represent the significance of the correlation in the graph, we magnify the coefficients and the 95% confidence intervals by a factor of 100, respectively.

Abbreviations: Mortality rates I, Mortality rates from Acute Myocardial Infarction; Mortality rates II, Mortality rates from Heart Failure; Mortality rates III, Mortality rates from Pneumonia in Children; Mortality rates IV, Mortality rates from Coronary Artery Bypass Grafting; Complications IV, Complication rates from Coronary Artery Bypass Grafting; LoS I, length of stay from Acute Myocardial Infarction; LoS II, length of stay from Heart Failure; LoS III, length of stay from Pneumonia in Children; LoS IV, length of stay from Coronary Artery Bypass Grafting; Satisfaction I, Satisfaction of outpatients; Satisfaction II, Satisfaction of inpatients; Satisfaction III, Satisfaction of medical staff.

Table S3. Heterogeneous Analysis of Hospital Performance and Overall Management Scores across Quartiles

	(1)	(2)	(3)	(4)	(5)	(6)	(7)	(8)	(9)	(10)	(11)	(12)	(13)
	AMI		HF		PC		CABG			Satisfaction ratings and turnover			
Dependent variable	Mortality rates	LoS	Mortality rates	LoS	Mortality rates	LoS	Mortality rates	Complication rates	LoS	Outpatients	Inpatients	Medical staff	Staff turnover
Panel A: low quartile (1/3)													
Overall management score	0.08<br>(0.56)	-0.03<br>(0.23)	0.25<br>(0.25)	0.24<br>(0.27)	-0.17<br>(0.21)	-0.00<br>(0.23)	0.83<br>(1.02)	0.82<br>(4.77)	-0.33<br>(2.64)	-0.93<br>(0.83)	-0.25<br>(0.40)	-0.21<br>(0.89)	0.01<br>(0.01)
N	146	146	146	146	145	145	89	89	89	164	167	168	172
R2	0.75	0.72	0.77	0.74	0.68	0.74	0.95	0.86	0.85	0.37	0.44	0.41	0.33
Mean values of the outcome	5.50	9.40	2.41	9.85	0.32	7.95	2.63	6.37	25.90	82.40	89.78	74.72	0.03
Panel B: middle quartile (1/3)													
Overall management score	-0.48<br>(0.53)	-0.22<br>(0.35)	0.00<br>(0.35)	-0.19<br>(0.27)	0.03<br>(0.04)	-0.05<br>(0.22)	0.87<br>(2.56)	3.21<br>(7.17)	-5.03<br>(7.94)	0.70<br>(0.60)	0.91<br>(0.65)	0.72<br>(1.05)	-0.01*<br>(0.01)
N	148	148	148	148	148	148	99	99	99	163	163	161	169
R2	0.84	0.69	0.72	0.73	0.75	0.75	0.71	0.68	0.67	0.44	0.43	0.44	0.42
Mean values of the outcome	4.99	9.24	2.59	9.50	0.08	7.21	2.35	2.32	26.78	83.13	90.47	75.05	0.03
Panel C: high quartile (1/3)													
Overall management score	-1.13*<br>(0.43)	-0.10<br>(0.26)	-0.64**<br>(0.22)	-0.32<br>(0.23)	0.01<br>(0.01)	-0.34*<br>(0.14)	-1.66<br>(0.98)	-6.68**<br>(2.10)	-3.41<br>(2.03)	1.20<br>(0.99)	0.98*<br>(0.45)	1.87<br>(1.01)	-0.01*<br>(0.00)
N	161	161	161	161	160	160	104	104	104	162	163	167	168
R2	0.91	0.81	0.76	0.77	0.99	0.88	0.77	0.89	0.88	0.40	0.62	0.59	0.43
Mean values of the outcome	5.79	9.61	2.31	9.64	0.07	7.49	2.07	3.80	26.96	82.76	90.30	75.80	0.02

Notes: The data is from the GHMS-China. ** and * represent significance at the 1% and 5% level, respectively. Standard errors are clustered at the hospital level. Abbreviations: AMI, Acute Myocardial Infarction; HF, Heart Failure; PC, Pneumonia in Children; CABG, Coronary Artery Bypass Grafting; LoS, length of stay in the hospital.
